# A heterogeneous artificial stock market model can benefit people against another financial crisis

**DOI:** 10.1371/journal.pone.0197935

**Published:** 2018-06-18

**Authors:** Haijun Yang, Shuheng Chen

**Affiliations:** 1 School of Economics and Management, Beihang University, Beijing, China; 2 Beijing Advanced Innovation Center for Big Data and Brain Computing, Beihang University, Beijing, China; 3 Department of Economics, National Chengchi University, Taipei, Taiwan, R.O.C; Central South University, CHINA

## Abstract

This paper presents results of an artificial stock market and tries to make it more consistent with the statistical features of real stock data. Based on the SFI-ASM, a novel model is proposed to make agents more close to the real world. Agents are divided into four kinds in terms of different learning speeds, strategy-sizes, utility functions, and level of intelligence; and a crucial parameter has been found to ensure system stability. So, some parameters are appended to make the model which contains zero-intelligent and less-intelligent agents run steadily. Moreover, considering real stock markets change violently due to the financial crisis; the real stock markets are divided into two segments, before the financial crisis and after it. The optimal modified model before the financial crisis fails to replicate the statistical features of the real market after the financial crisis. Then, the optimal model after the financial crisis is shown. The experiments indicate that the optimal model after the financial crisis is able to replicate several of real market phenomena, including the first-order autocorrelation, kurtosis, standard deviation of yield series and first-order autocorrelation of yield square. We point out that there is a structural change in stock markets after the financial crisis, which can benefit people forecast the financial crisis.

## Introduction

Advances in computing had an immense influence to finance and economics. Today, there is a huge amount of financial data available for analysis, and we also have such strong processing power to analyze these data. It stimulates us to find new routes for studying finance and economics, to get a better understanding of the behavior of financial markets, and even opens brand new areas of research in this field [1,2]. Furthermore, agent-based models can help financial regulators to predict and prevent meltdowns of financial markets [3]. Likewise, agent-based computational finance can provide a unique understanding of the emergent properties of financial markets where instinct fails [4,5]. Although, they neglect the difference between advanced and emerging financial markets.

Despite the research on the macro factors to evaluate the impact of financial stability [6,7], little exists in the literature to assess the impact of financial crisis on the microstructure by agent-based models (ABM) [8]. The financial market is a particularly good application for ABM method. The Santa Fe Artificial Stock Market, SFI-ASM, is one of the most successful attempts using ABM to do research on the financial market. It has formed a platform, which is outlined and developed by Arthur et al. [9]. The market structure derives from the work of Bray [10] and Grossman et al. [11]. In this framework, agents which are myopic and have constant absolute risk aversion utility chose between a risk free bond and a risky stock paying a stochastic dividend. This model aims to understand what kind of market features would emerge after long time process of learning and adaptation of heterogeneous agents. However, their models assume that all agents are homogeneous to share the same fundamental information, which will be ameliorated in this paper.

Through continuing developments, there are several aspects of productions based on SFI-ASM. Some researchers try to modify the evolution algorithm. Tay and Linn [12] use a fuzzy logic system to replace the original classifier forecasting system and change the style which agents used to choose a forecasting strategy in demand decision. Chen and Yeh [13] add a social learning mechanism—a public pool of evolving strategies; so agent not only studies from the historical information about itself but also from the public pool. Agents compare their current wealth with others’ and balanced their behavior during the latest period to decide how to update their strategies by public pool. But they just focus on the simulation results and ignore the real financial markets, which will be overcome in this paper.

Some simulations have focused on empirical features of real markets. LeBaron et al. [14] describe the different features of time series produced by ASM when using different implementation frequency of genetic algorithm. Setting low learning speed, the market converged to the rational expectations equilibrium where all agents have homogeneous agreement on processing the fundamental dividend information; the market price well presents the asset value and experimental data shows low price fluctuation and low trading volume. Setting high learning speed, the statistical features of market price and volume are very close to the real data of the financial market. Beltrametti et al. [15] research on evolving and forecasting issues in exchange market which introduces real data for model training and validating. The experimental results display that the accuracy of model forecasting is lower than VAR model in Dollar-Mark market, but higher in Dollar-Yeh market. Navarro and Larralde [16] reproduce an asymmetry in the distribution of losses and gains in financial market using an agent based model of a single asset.

Some researchers have improved the structure of the model. Pascual et al. [17] propose the factors of mentality and sentiment of agents in their model. The statistical features of experimental data are compared with Spain Ibex-35 index. The modified model is prior to the original one. Ehrentreich [18] indicates the impact of learning speed and size of active strategy pool to the wealth level of agents. He suggests fixing the size of the pool as it always gets smaller through the time. Otherwise, 50 stocks in NYSE are sampled as a benchmark; an artificial stock market model is proposed to validate the characters of security returns [19]. Using leveraged funds, heavy tail and clustered volatility appeared which could be observed in real financial markets. The reason is the risk control policy of the banks in their model [20]. Their works all implicate that experimental results are consistent with the observed character of real financial market data. Furthermore, heterogeneous and informed agents are used to frame an artificial stock market, which focuses on the statistical properties of price and return to test some multivariate stylized facts [21].

And there are many other aspects of works based on SFI-ASM. Joshi et al. [22] explore the interactions between the technical and fundamental agents. The result shows that using technical trading bits is a dominant strategy in the market. Whether all other traders are using technical bits or fundamental ones, it is an optimal strategy for the new agent to add technical bits. The existing of trend-following behavior may be lasting in a market. They believe the extensively using of technical bits weakened the ability of forecasting and magnified the volatility of market price. Bertella et al. [23] investigate the dynamics of stock price fluctuations and return in an artificial financial market which includes fundamentalist and chartist agents with and without confidence. They point out that that stock price significantly affects confidence index, but not vice versa.

Although the SFI-ASM is an excellent platform for further studies, there are some flaws. One of the deficiencies is the homogeneity of agents. All of the agents are rational investors. They are endued with same the function of utility, speed of studying, size of strategy pool and get the same information from financial markets. Although they produced different strategies through evolving process and made different decisions based on their own pool of strategies, the design pattern of homogeneity brought homogeneity evolving results of strategies and finally gave macro phenomenon of low level of price volatility, closing to zero trading volume and similar agent’s wealth, which were not coherent with the real world.

This article attempts to improve the SFI-ASM system by adding heterogeneity of agents to make it better fit with the statistical features of data produced by the real world. The model is comprised of zero-intelligent agents, less-intelligent agents, and intelligent ones. The heterogeneity of agents is presented by the level of intelligence, the evolving speed, the pattern of information used, the size of strategy pool, the rules for a strategy to be active and the aversion level of risk. The experiments are completed to probe the parameters that enable the modified model to perform steadily and the proportion of different kinds of agents that better simulate the real market features than the original model.

ASM offers a new routine to research on the reality features emergent in financial market and complex market operational mechanism. So it has theoretical and practical significance to make ASM more close to the real financial market. This article wants to achieve it by further understanding and redefining of agents in ASM system.

The main contributions of this paper are as follows:

- On the basis of SFI-ASM, a modified model is proposed to suit the real financial markets by adding zero-intelligent and less-intelligent agents.
- The crucial parameter has been found to keep the system steadily. The upper bound of the maximum percentage of zero-intelligence and less-intelligence could be added to model is 33.3% and 16.7% respectively in the system.
- Agents are divided into different learning speeds, strategy-sizes, utility functions, and level of intelligence. According to different financial market, the statistical features of data are more close to those of the real world.
- By calculating the first-order autocorrelation, kurtosis, standard deviation of yield series and the first-order autocorrelation of yield square of real financial market data, an optimal model after the financial crisis is validated. Furthermore, we find the structural change in stock markets after the financial crisis.

The remainder of this paper is structured as follows. Section 2 presents the modified model. Section 3 provides a concrete experimental design followed with the experiment results and econometric analysis. In section 4, the optimal models before and after the financial crisis are offered. Results are summarized and discussed in section5.

## Frame of the model

In this section, the environment of the artificial stock market, anticipants, forecasting method and modified model will be introduced one by one. All the values of parameters mentioned in this section will be presented in next section (Table 1).

**Table 1:** Values of main parameters of the model.

p-crossover	0.3	interest rate(r)	0.1
p-random	0.333	lambda	0.5
p-linear	0.333	Number of forecasts	100
p-mutation	0.03	baseline	10
Evolving speed	250	Number of agents	30
sigma	0.4	minexcess	0.01

### Environment

There are two financial assets in the artificial stock market. A risk-free asset provides a constant net return rate and a risky asset pays an uncertain dividend *d* at the end of each period. The dividend is denoted as following:

$$d_{t}=\text{baseline}+\rho (d_{t-1}-\text{baseline})+\varepsilon_{t}$$ (1)

Where *ε*_*t*_ obeys Gaussian distribution with zero mean; *ρ* and *baseline* are constants.

The current market state is denoted as a *J*-bit array. According to the current market state, if the *kth* description is true, the bit in position *k* will take on the value 1 (01 in binary). On the contrary, the bit in position *k* will take on the value 2 (10 in binary).

### Anticipants

Market participants comprise a market maker and *N* stock market traders (agents). Each agent has the same shares of the stock and same positive wealth in initial. Agents share identical utility of wealth function *U*(*W*),

U(W)=−exp(−lambda*W) (2)

At the beginning of each period, agents make a portfolio to maximize expected utility of next period, *Max*(*E*(*U*(*W*_*t*+1, *i*_)) subjects to the condition as below:

$$W_{t+1,i}=x_{t,i}(p_{t}+d_{t})+(1+r)(W_{t,i}-x_{t,i}p_{t})$$ (3)

Where *r* is risk-free rate; stock demand of agent *i* is denoted as *x*_*t*,*i*_; *p*_*t*_ is the stock price.

It is well known that the optical demand to maximize the utility of agent is given by (Arthur et al. 1997)

$$x_{t,i}=\frac{E_{t,i}(p_{t+1}+d_{t+1})-p_{t}(1+r)}{lambda*\sigma_{t,i,p+d}{}^{2}}$$ (4)

The market maker will congregate the demand information and get a market clearing price for the stock in turn. Then it transmits this clearing price to each agent. If the bid and ask price spread is large, the market maker will adjust the trial-price a little by a specific process and then send trial-price back to agents to college the bid-ask information again. This process will repeat until the imbalance is smaller than a threshold value which is denoted as “minexcess” or certain iterative times is achieved.

### Forecasting method

The agents use Eq 5 to forecast the stock price.

$$E(p_{t+1}+d_{t+1})=a(p_{t}+d_{t})+b$$ (5)

In order to get optimal forecasts, agents employ a list of *K* candidate forecasting rules. These rules maps market states into parameters— *a* and *b* for forecasts, which is denoted if-then forecasting rules. Each forecast variance of rules forms as a weighted average of the rule’s past squared forecast errors as Eq 6.

$${\text{var}}_{t,i,j}^{2}=(1-\frac{1}{\theta }){\text{var}}_{t-1,i,j}^{2}+\frac{1}{\theta }{\left[(p_{t}+d_{t})-E(p_{t}+d_{t})\right]}^{2}$$ (6)

Agents use genetic algorithms to filtrate forecasting rule. The value of fitness is given by Eq 7, which is calculated by its forecast variance and specificity which counts down the specific condition bits in its condition statement.

$$fitness_{t,i,j}=-{\mathrm{var}}_{t,i,j}-bit\cos t*specificity_{t,i,j}$$ (7)

Based on the fitness, each agent independently updates his set of active forecasting rules by genetic algorithms. The learning process of each agent runs independently with a certain probability *Pu* at the end of each trading period. The worst-performing rule is deleted from the set. They will be replaced by new rules generated by genetic operators [5].

### Modified model

Our model is completed by Objective-c and the Swarm version of ASM is 2.4 releases. The modified model focuses on following three aspects:

#### 1) Matching rule and the size of active strategy pool

The active strategy is the strategy which matches the current market states. All active strategies form the active strategy pool, which is a subset of agent’s strategies. The Agent chooses optimal forecasting rule to compose the active strategy pool, and the size of it has a crucial influence on the accuracy of forecasting markets and evolutionary quality of strategy. The matching rule of original version requests all bit-unit matching, and asks only any bit-unit matching in recent version considering computing speed. This article introduces a new variable “suit” to record the amount of bit-unit matching the current market state and estimates the matching degree of forecasting rule.

To avoid the size of active pool getting too small through the time, it is fixed in later experiments. All rules are arrayed on “suit”, and the part with larger “suit” is chosen as the set of active strategy.

#### 2) The optimal strategy

The rules evolve based on fitness, according to formula (8), which implicates rules with lower forecasting error and better versatility has a high probability to survive.

choice=sigma*var+(1−sigma)*(−suit) (8)

We use the past performance of a rule to measure the variable “var”, and apply the matching degree between the rule and the current market status to denote the variable “suit”. This modified style of choosing better reflects the situation of the real financial market. When estimating a rule, agents need trade-off the performance between past and now. So they not only take into account the forecasting accuracy of this rule in past time, but also consider how it is fitting with the current information of the financial market. The rule of the lowest value of variable “choice” is chosen as the optimal rule for forecasting of next step.

#### 3) Monitor of the heterogeneous market information

In the original model, the whole market information is comprised of 61 bit-units. All agents choose the same 12 bit-units from the whole market information vector as their fixed states for monitoring. Considering the real world, traders are generally interested in different parts of large mount market information. Thus, changing the pattern as following, each agent chooses its own 12 bit-units’ vector forming its own view about the market. Their vectors of market information become different. Agents may emphasize on technical bit-unit or fundamental bit-unit, instead of using fixed numbers of technical and fundamental ones. This amelioration is essential, which makes agents are heterogeneous in the financial markets.

## The Design of Experiments

The basic values of main parameters are exposed as below [9, 24]. If not mentioned, the values of parameters listed in Table 1 are used in below experiments. However, some of them are set different for specific experiments.

Where p-crossover is the probability of crossover operator; p-random is the probability of selection of parents; p-linear is the probability of the weighted value of linear correlation of parents; p-mutation is the probability of mutation operator; minexcess is the threshold value of spread of bid and ask price.

### Real data sampling and original model comparison

The real market data are chosen as the benchmark. All of the chosen data are the important stock index of China financial market. The detailed information of the data is as follows. Shanghai Composite Index (code: 000001) released at 1990/12/21. Our experiment intercepts the data from 1994/01/03 to 2008/09/18 and omits the ones before 1994 by considering them as the trial data. Shenzhen Component Index (code: 399001), Shanghai A-Circulating Shares market value weighted Index (code: 1120001), Shenzhen A-Circulating Shares market value weighted Index (code: 2120001), all of them are intercepted the same period of time as that of Shanghai Composite Index.

The frequency of the data is set as day and week, the first-order autocorrelation, kurtosis, standard deviation of yield series and the first-order autocorrelation of yield square (cluster volatility) are calculated and recorded as follows:

The statistical features of the original model are as follows. *K* denotes the evolving speed, the time interval between observations is denoted as *I*, *I* = 1 is the daily frequency of trading data and *I* = 5 is the weekly frequency of trading data. Each experiment executes five times by different random seeds and the average of the data is taken as below. For each experiment, the market runs for 250000 time periods in order to allow sufficient learning, and get rid of early abnormal fluctuations. Then next 1000 time series of samples are recorded.

The Tables 2 and 3 show that there is an obvious difference in the statistical features between real market data and the ones produced by original ASM model. As for yield series, at 95% confidence level, there is no first-order auto-correlation in Shanghai index at any frequency and in Shenzhen index at the weekly frequency; and there is little auto-correlation in Shenzhen index at daily frequency. However, for both evolving speeds, the original ASM model produces large negative auto-correlation at the weekly frequency (*I* = 5). As for the kurtosis, all of the real data possess much larger than the experimental data, and kurtosis is much larger at the weekly frequency than the daily frequency in real data. As for standard deviation, the ASM model performs well. The square of yield series possesses positive first-order auto-correlation at the daily frequency of real market data while it has no auto-correlation at the weekly frequency. But the data of ASM presents little first-order auto-correlation at daily frequency. For weekly frequency, the experimental data show different performances, it has large positive auto-correlation when evolving speed is slow and little auto-correlation when the speed is faster.

**Table 2:** Statistical results of real stock index.

Code	000001(day)	1120001(day)	399001(day)	2120001(day)
autocorrelation	0.006 (p = 0.705)	0.014 (p = 0.398)	0.043 (p = 0.010)	0.053 (p = 0.002)
Kurtosis	36.374	27.051	14.654	19.003
Std.Dev	0.0224	0.0232	0.0216	0.0230
Square -auto	0.115 (p = 0.000)	0.172 (p = 0.000)	0.125 (p = 0.000)	0.124 (p = 0.000)
Code	000001(week)	1120001(week)	399001(week)	2120001(week)
autocorrelation	0.004 (p = 0.911)	0.003 (p = 0.937)	0.061 (p = 0.098)	0.011 (p = 0.757)
Kurtosis	146.171	389.332	16.247	515.687
Std.Dev	0.0582	0.1024	0.0493	0.1324
Square -auto	0.006 (p = 0.864)	-0.002 (p = 0.956)	0.052 (p = 0.153)	-0.002 (p = 0.962)

**Table 3:** Statistical results by original model.

	K = 1000 I = 1	K = 1000 I = 5	K = 250 I = 1	K = 250 I = 5
autocorrelation	0.088	-0.192	-0.027	-0.201
Kurtosis	3.107	4.807	3.218	3.210
Std.Dev	0.024	0.082	0.028	0.075
Square -auto	0.039	0.286	0.027	0.154

Using the modified structure of ASM anterior mentioned, the below experiments try to better simulate the real market data by appending heterogeneous to agents from different aspects.

### The market of intelligent agents

In this subsection, agents with full level intelligence are divided into different learning speeds, strategy-sizes and utility functions.

### Agents with different evolving speed

Evolving speed is denoted as *K*, which taking *K* generations to perform genetic algorithms. The agents are divided into three different evolving speeds, the slow speed (*K* = 1000), the middle speed (*K* = 250), the fast speed (*K* = 100). The percentage of agents with specific evolving speed is an important parameter in this experiment. Five kinds of percentage vectors are set in advance. Ten runs with different random seeds are done for every vector before data are sampled at daily frequency, and then other five runs for weekly frequency. The vector is assigned as (fast, middle and slow).

Comparing with real data, when using parameters vector (2) and (5), Table 4 shows that the yield series presents no first-order auto-correlation, and the square of yield series exhibits large positive first-order auto-correlation which the original model does not show, well fitting the statistical features of real financial market data at daily frequency. But they have little larger standard deviation value. All of the experimental data has no improving in the value of kurtosis; the experimental results are much smaller than the real ones.

**Table 4:** Statistical results of daily frequency.

I = 1	1: 1: 1	1: 2: 2	2: 2: 1	1: 3: 2	1: 3: 1
Autocorrelation	-0.082	-0.035	-0.357	-0.553	-0.041
Kurtosis	3.342	3.672	5.877	9.289	4.402
Std.Dev.	0.0323	0.0238	0.0453	0.1455	0.0377
Square -auto	0.100	0.160	0.318	0.778	0.132

The results of experiment (Table 5) are not good enough comparing with real data. The vector (2) shows some level of first order auto-correlation about yield series which is smaller than original model, and the first order auto-correlation about square of yield series is also smaller than that of original model. The standard deviation is closer to that of real data. But, the value of kurtosis is still too small.

**Table 5:** Statistical results of weekly frequency.

I = 5	1: 1: 1	1: 2: 2	2: 2: 1	1: 3: 2	1: 3: 1
Autocorrelation	-0.100	-0.106	-0.296	-0.137	-0.284
Kurtosis	3.488	3.978	77.811	6.934	6.341
Std.Dev.	0.0490	0.0488	0.9852	0.0587	0.0789
Square -auto	0.107	0.055	0.162	0.107	0.073

### Agents with different sizes of strategies

Agents are divided into three different strategy sizes, the big size (Fnum = 300), the middle size (Fnum = 100) and the small size (Fnum = 30). The vector of percentage is assigned as (big, middle and small). It is a key parameter in this experiment. Five different kinds of the vector are predefined. Ten runs with different random seeds are selected for every vector in daily frequency and weekly frequency respectively, and the average results are recorded as follows:

For daily frequency, Table 6 shows that there is not the first-order autocorrelation about yield series and vector (2) and (5) present autocorrelation of the square of yield series. For weekly frequency, all vectors of Table 7 show the first-order autocorrelation of yield series, and the square of yield series show little autocorrelation than that of the original model. However, the kurtoses are still too small compared with that of real data for both situations.

**Table 6:** Daily frequency with different strategy-size.

I = 1	1: 1: 1	1: 1: 3	3: 1: 1	2: 2: 1	1: 2: 2
Autocorrelation	0.004	-0.025	-0.09	0.046	-0.054
Kurtosis	3.172	3.953	2.952	3.143	3.501
Std.Dev.	0.0272	0.0248	0.0233	0.0255	0.0324
Square -auto	0.058	0.112	0.075	0.072	0.105

**Table 7:** Weekly frequency with different strategy-size.

I = 5	1: 1: 1	1: 1: 3	3: 1: 1	2: 2: 1	1: 2: 2
Autocorrelation	-0.112	-0.123	-0.277	-0.168	-0.101
Kurtosis	4.103	3.920	4.752	3.795	3.176
Std.Dev.	0.0803	0.0704	0.0842	0.0825	0.0921
Square -auto	0.044	0.098	0.110	0.078	0.077

### Agents with changing utility function

Considering the mentality of agents, they are offered with various risk aversions. The agents are still using CARA utility function, but the parameter of the function is changing according to the wealth level of agent in current time [25]. If the wealth of agent is larger than the average wealth of all agents during the last ten periods, the agent feels that it is successful in previous time and could endure higher risk level. It reduces its risk aversion parameter—lambda. If the wealth is no longer above average level, then it returns its lambda to original value. In experiments, the low level lambda is set to be 0.3 and the high level is set to be 0.7 in experiments.

This experiment divides agents into two kinds: the agents of the original model and the agents mentioned just above with changing utility function. The vector of percentage is set as (normal: changed). As above, five different kinds of this vector are predefined. Ten runs with different random seeds are used for every vector in daily frequency and weekly frequency respectively, and the average results are recorded as Tables 8 and 9.

**Table 8:** Daily frequency with changing utility function.

I = 1	1: 1	1: 2	2: 1	7: 23	23: 7
Autocorrelation	0.004	-0.001	0.021	0.004	0.031
Kurtosis	6.109	8.423	6.004	13.119	7.443
Std.Dev.	0.0282	0.0226	0.0215	0.0212	0.0289
Square -auto	0.098	0.110	0.154	0.106	0.152

**Table 9:** Weekly frequency with changing utility function.

I = 5	1: 1	1: 2	2: 1	7: 23	23: 7
Autocorrelation	-0.153	-0.175	-0.2	-0.229	-0.139
Kurtosis	6.987	7.356	6.442	9.283	6.040
Std.Dev.	0.0851	0.0825	0.0958	0.100	0.0832
Square -auto	0.129	0.071	0.05	0.096	0.045

At daily frequency, all vectors show no first-order autocorrelation of yield series and autocorrelation about squared of yield series, just like real data. For weekly frequency, all vectors show the first-order autocorrelation of yield series much larger than that produced by real data. All vectors show little autocorrelation squared of yield series than the original model. Hereinto, vector (3) and vector (5) present no autocorrelation. For both frequencies, the kurtoses are larger than the value of the original model, though still smaller than the value of real data.

### Summary

The representative real market data produced by original model and the optimal modified models are summarized as Tables 10 and 11.

**Table 10:** The comparison of daily frequency.

	Auto-correlation	Kurtosis	Std.Dev.	Square–auto
000001	0.006	36.374	0.0224	0.115
Original model K = 1000 I = 1	0.088	3.107	0.0240	0.039
Original model K = 250 I = 1	-0.027	3.218	0.0281	0.027
Different evolving Speeds 1: 2: 2	-0.035	3.672	0.0238	0.160
Different strategy Size 1: 1: 3	-0.025	3.953	0.0248	0.112
Changing utility Function 1: 2	-0.001	8.423	0.0226	0.110

**Table 11:** The comparison of weekly frequency.

	Auto-correlation	Kurtosis	Std.Dev.	Square–auto
000001	0.004	146.171	0.0582	0.006
Original model K = 1000 I = 5	-0.192	4.807	0.082	0.286
Original model K = 250 I = 5	-0.201	3.210	0.075	0.154
Different evolving Speeds 1: 2: 2	-0.106	3.978	0.0488	0.055
Different strategy Size 1: 1: 1	-0.112	4.103	0.0803	0.044
Changing utility Function 23: 7	-0.139	6.040	0.0832	0.045

At daily frequency of Table 10, the optimal of all the three experimental data of modified model is consistent with the representative real data in autocorrelation of yield series and squared of yield series. About weekly frequency of Table 11, the optimal of all the three experimental data possess a lower level of the first-order autocorrelation of yield series than that of the original model. The data of second and third experiments well fit the real data of squared of yield series; data of first experiment present little of it. The data of changing utility function bring much larger kurtosis, but still smaller than that of real data.

### The market of agents with different level of intelligence

In real financial markets, not all the traders are completely rational or have enough information to make decisions. Many traders always puzzled by likely situations, such as without entire information, or not making optimal decisions based on full information from the past. So, this article adds two kinds of agents with different intelligent level, the zero-intelligent agents and the less-intelligent agents, into the original model in order to well simulate the real market. An intelligent agent has the ability to choose optimal forecasting rule in active strategy pool and improves its ability by continuing evolution. And this is main design discrepancy with zero-intelligent agents and less-intelligent ones.

The zero-intelligent agents cannot evolve in the system; neither can they choose the optimal solution for forecasting. They even do not reserve a set of forecasting rules. The forecasting rule is created by valuing its parameters randomly within certain region instead of choosing from an evolving rule set. The demand for the risky asset is still calculated based on maximizing utility function and the forecasting error is measured by the difference between forecasting price and the last period one.

The less-intelligent agents have the capacity to evolve in the system, but they cannot choose the optimal solution from active strategy pool with reserving a set of forecasting rules. This kind agent has the ability to observe the past forecasting errors of a rule (phenotype of a rule), but it cannot master a rule which should be used in different situations by lacking information or intelligence (genotype of a ruler). The forecasting rule set of an agent contains rules performed well in past time and updates them based on a weighted moving average of performance. When a new situation arises, agents do not know how to choose the optimal forecasting rule to well match this kind of state, so it can only choose one by random.

### The results of original model

The below experiments append zero-intelligent agents and less-intelligent ones to original model respectively. The parameter “Num” denotes the number of zero or less intelligent ones in the model. Five runs with different random seeds are completed for every parameter at a daily frequency and weekly frequency respectively. After 250000 time periods, the next 1000 samples are recorded. The average of price, standard deviation, the percentage of price reaching the lowest point of the model (it set to be 0.001) and the percentage of price exceeding 200 are summarized as Tables 12 and 13.

**Table 12:** Zero-intelligent agent for weekly frequency.

Num	1	2	3	4	6
Price	68.01	27.02	27.78	2.44	2.13
Std.Dev	6.87	4.71	4.98	3.76	3.53
Reaching 0.001	0	12.1%	34.9%	48.3%	55.6%
Exceeding 200	0	0	0	0	0

**Table 13:** Less-intelligent agent for weekly frequency.

Num	1	2	3	4	6
Price	1.49	2.82	5.91	60.73	98.87
Std.Dev	1.92	3.12	11.20	205.88	426.43
Reaching 0.001	28.9%	34.2%	51.0%	38.2%	9.8%
Exceeding 200	0	0	0	0.5%	3%

The representation of daily frequency is similar. The experiment shows that direct adding of zero and less-intelligent agents brings damaging influence on the original model. The value of price is abnormality: a wide range of minimum value emerges in all experiments. Increasing the zero-intelligent agents, we find that price reaches the minimum value with high probability and the average of price is low; we also discover that there is no abnormal high price appearance. Increasing the less-intelligent ones, we notice that volatility of price rises up significantly. The volatility is too large compared with the average price. Abnormal high prices emerge in some experiments, which magnify the volatility to a huge scale. That is to say, adding zero and less-intelligent agents in the model are the reason that experiments fail to simulate a real financial market. According to above results, this paper will modify some parameters or limitations to improve the adaptability of the model.

### The results of appending or modifying some parameters

For original model, there are trading limitations of total wealth and the maximum bid price of agents, but without minimum bid price. When the price reaches a certain high degree, the intelligent agents will reduce the amount of purchase and increase the sale volume, which makes the price drop, and vice versa. The model possesses a mechanism of stability. However, when the model contains zero-intelligent agents or less-intelligent ones, even if the price is raised to a considerable level, the random forecasting rules (zero-intelligent agents) and the random select forecasting rules(less-intelligent agents) can possibly guide them to make a purchase decision.

Moreover, without restriction of the smallest trading volume, agents perform purchasing under a finite amount of cash. So the trading pattern of high price and low volume make the price reach an abnormally high level, and vice versa. Without above restriction, some agents with limit cash have the willingness to buy. Thus, the price may be raised to an abnormally high level when high price and low trading volume of trading pattern appears. To overcome this problem, this paper adds a restriction of minimum trading volume for agents, denoted as “minbid”, which is set at 0.0001, 0.001 and 0.01, respectively.

The existing restriction of maximum trading volume in the original model has greatly impact on its price volatility. Agents have the capacity to carry out large trading volume at low price. Thus, too huge maximum biddings will cause large price volatility, and too small value of it may lead to producing smooth price sequences. In light of this, this paper resets the restriction of maximum trading volume “maxbid” to three values: 10, 5 and 1, respectively.

In addition, as mentioned above, the dealer adjusts price based on the imbalance volume between total purchase and total sale. The adjustment repeats until imbalance is smaller than “minexcess” or maximum iteration times is achieved. The value of “minexcess” also has an influence on price volatility. If the value of “minexcess” is easier to meet this condition, for imbalance, the dealer can formulate final price with fewer times of adjustment, which makes price process moving smoothly, and vice versa. Such that, this paper resets the threshold “minexcess” to four values: 0.1, 0.01, 0.001 and 0.0001, respectively.

The following experiments append a small amount of zero-intelligent agents (10%) or less-intelligent agents (10%) to the modified model, and then obtain appropriate parameters (“minbid”, “maxbid” and “minexcess”) to make the model perform steadily. Each experiment runs five times with different random seeds at a weekly frequency and daily frequency separately. The market is run for 250000 time periods in advance; then, next 1000 time series of samples are recorded.

If the experiment with some parameter combination performs well within 5 times, we extend the experiment to 10 times and lengthen the running periods to 100000 in order to support further verification.

#### 1) Maxbid = 10

**Fig 1:**
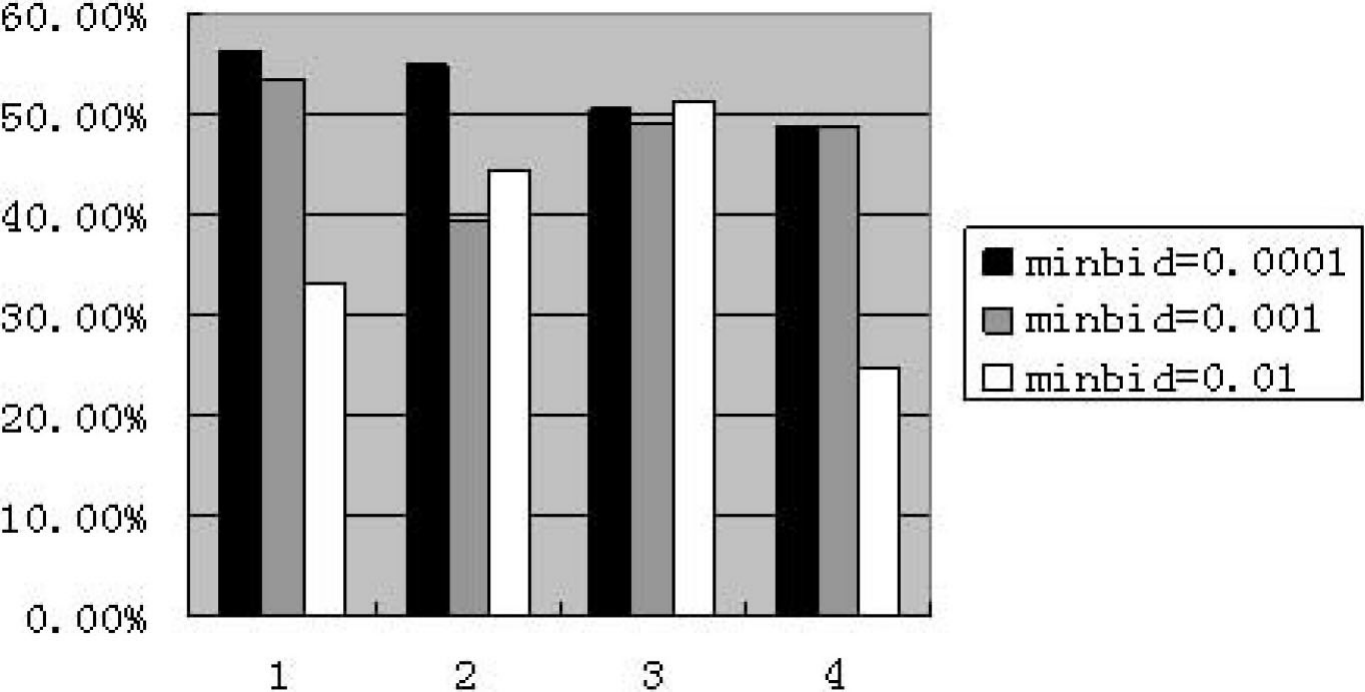
Percentage of reaching minimum value for less-intelligence at weekly frequency (maxbid = 10. The abscissa denotes four cases of “minexcess”; the ordinate denotes the percentage of price reaching the value of “minexcess”.).

As for market including zero-intelligent agents, the results of all experiments of different parameter combinations are bad. They show very low price, a wide range of minimum price, but without an abnormally high price. The combination of (minbid = 0.0001 and minexcess = 0.0001) presents good performance in only one experiment at the weekly frequency, but bad in other four times. The market including less-intelligent agents is similar: very low price, an even more wide range of minimum price without an abnormally high price. When setting “maxbid” as 10, the model performs poorly. The Fig 1 shows the percentage of price reaching the minimum value with 10% less-intelligent agents at the weekly frequency. The four categories of “minexcess” from left to right are 0.0001, 0.001, 0.01 and 0.1 respectively.

#### 2) Maxbid = 5

**Fig 2:**
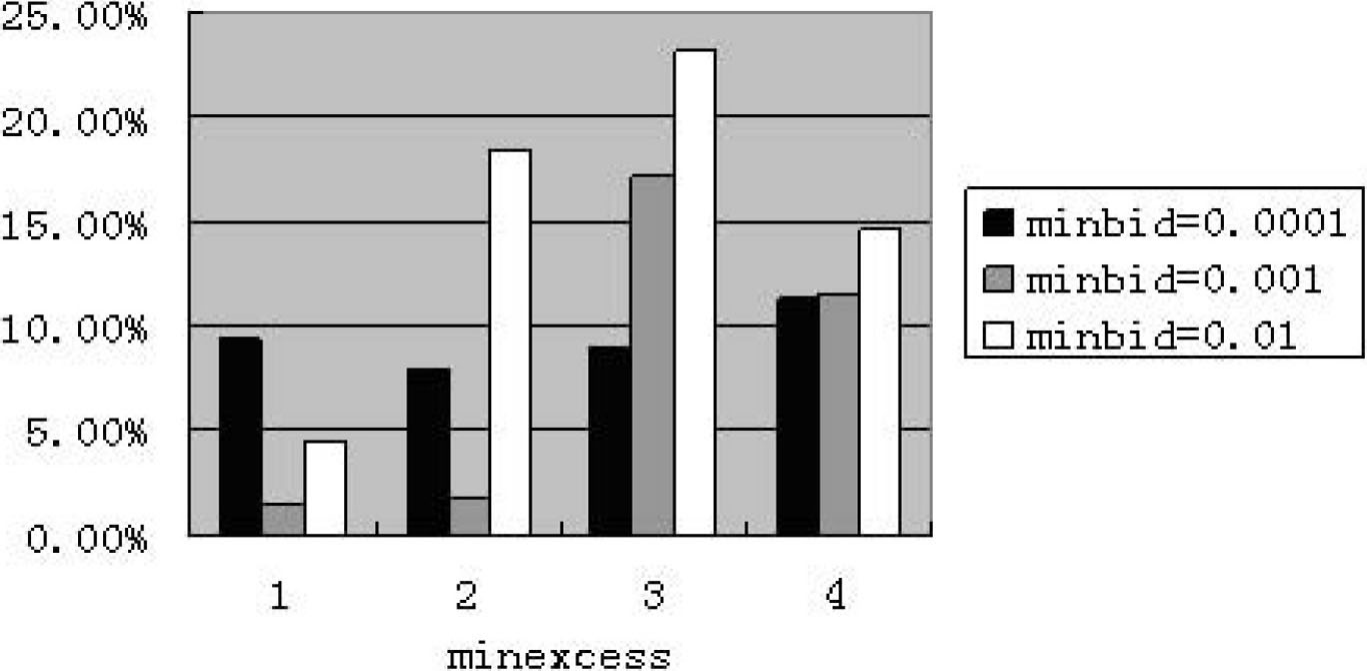
Percentage of reaching minimum value for less-intelligence at weekly frequency (maxbid = 5. The abscissa denotes four cases of “minexcess”; the ordinate denotes the percentage of price reaching the value of “minexcess”.).

**Fig 3:**
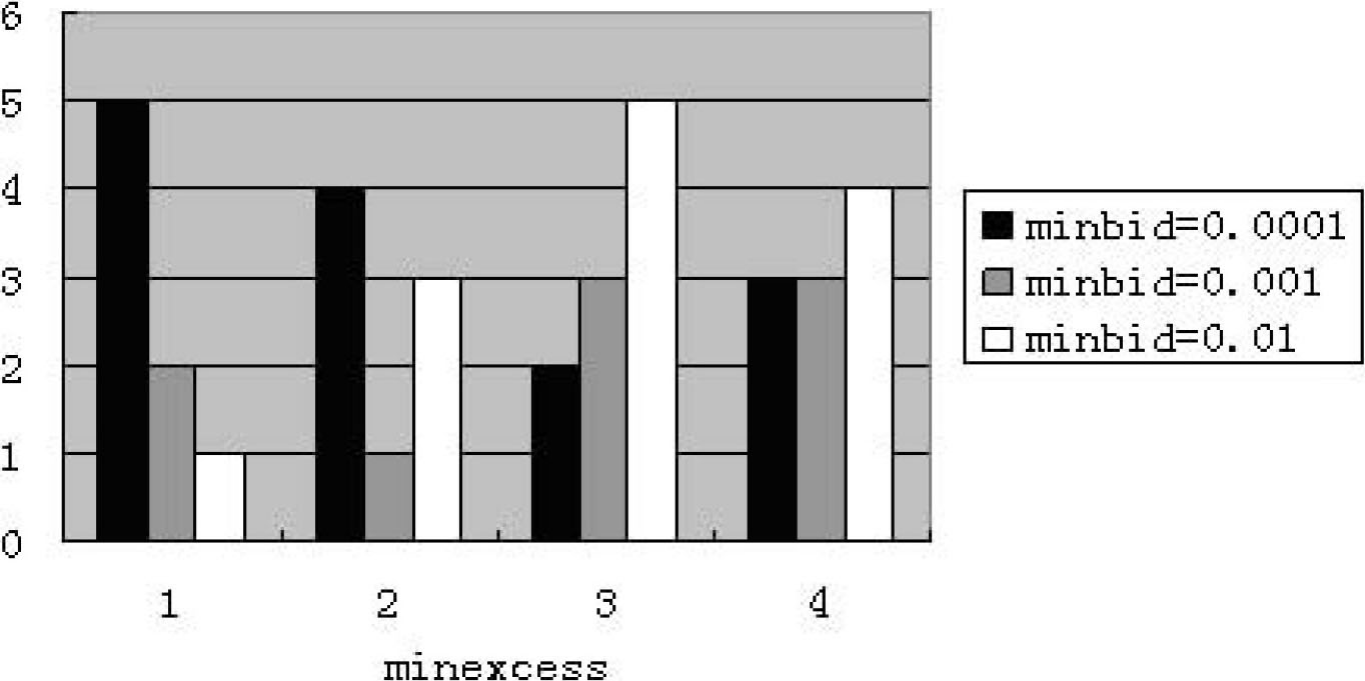
Experiments have minimum price appeared for zero-intelligence at weekly frequency (The abscissa denotes four cases of “minexcess”; the ordinate denotes the number of abnormal price.).

**Fig 4:**
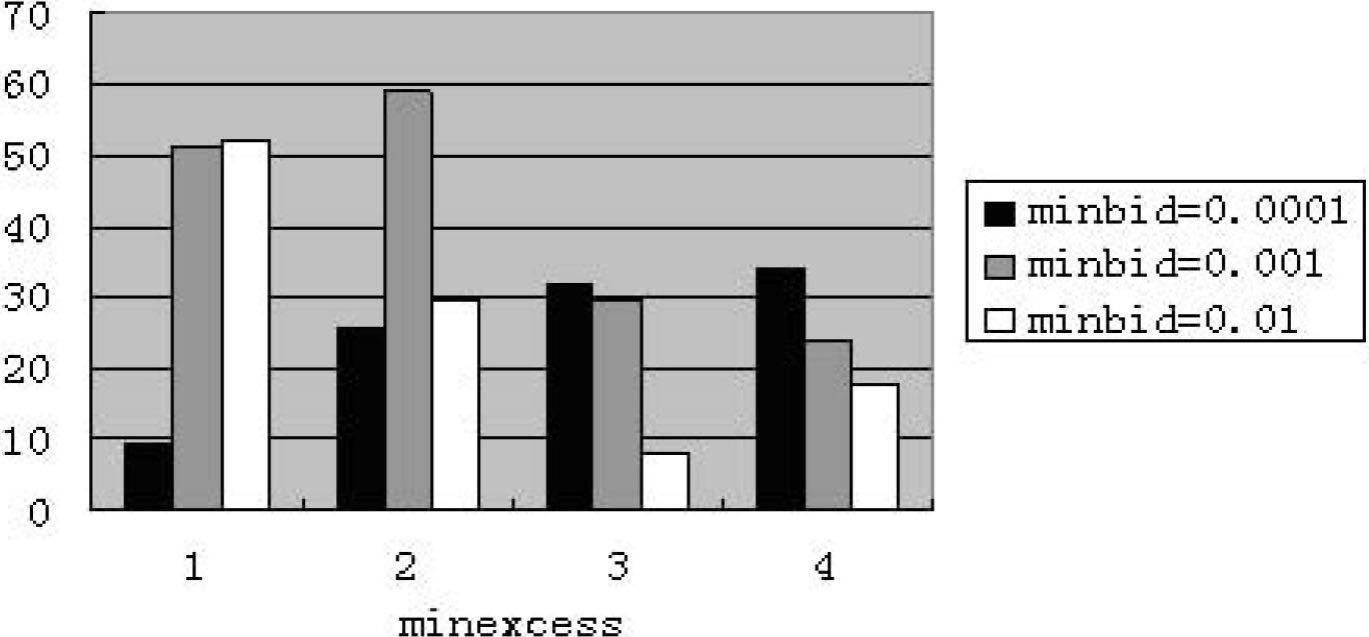
Average price for zero-intelligence at weekly frequency (The abscissa denotes four cases of “minexcess”; the ordinate denotes the stock price.).

When assigning “maxbid” to 5, we find that the performance including zero-intelligence is improved. The price becomes normal in some experiments by some random seeds. Fig 2 shows that average percentage of price reaches the minimum value. Fig 3 presents that the number of experiments with minimum value appears. Fig 4 illustrates the average price at weekly frequency. For above mentioned figures, the four categories of “minexcess” from left to right are 0.0001, 0.001, 0.01 and 0.1 respectively.

**Fig 5:**
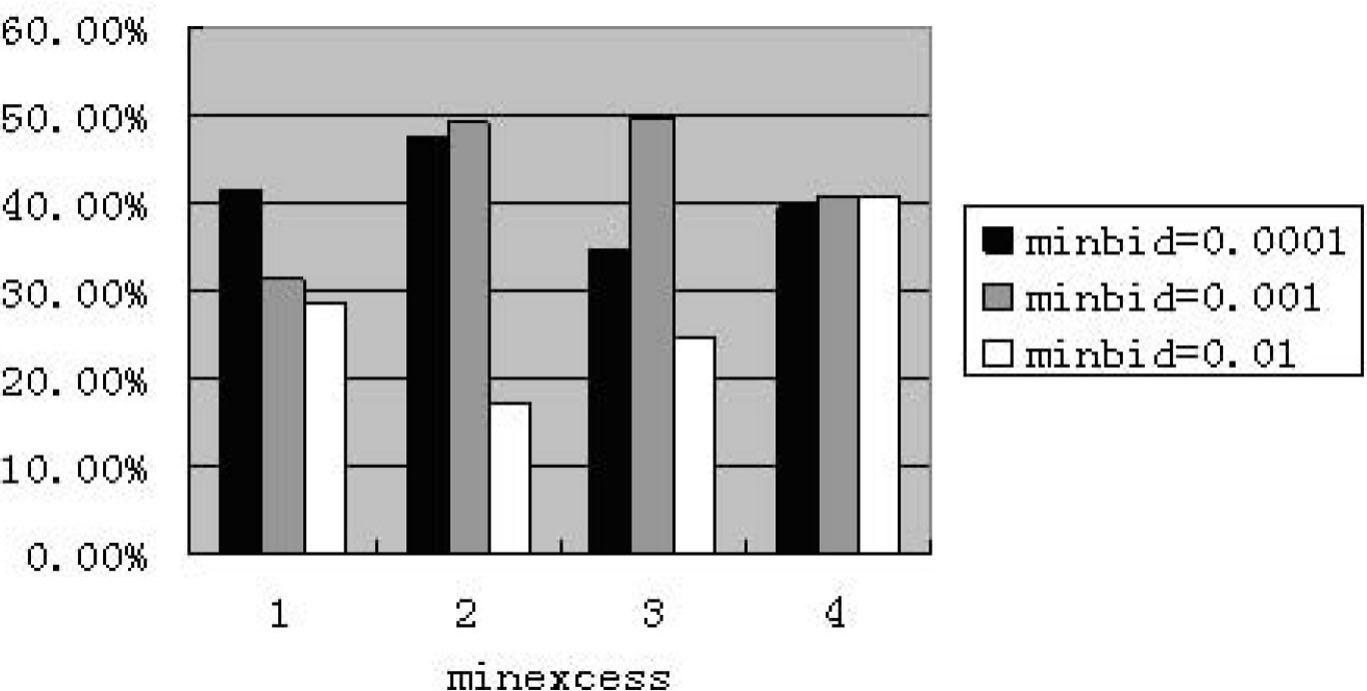
percentage of reaching minimum value for less-intelligence at weekly frequency (The abscissa denotes four cases of “minexcess”; the ordinate denotes the percentage of price reaching the value of “minexcess”.). P.

The performance of market including less-intelligence is still bad, and no experiment shows further improvement. The Fig 5 shows average percentage of price reaching the minimum value.

#### 3) Maxbid = 1

**Fig 6:**
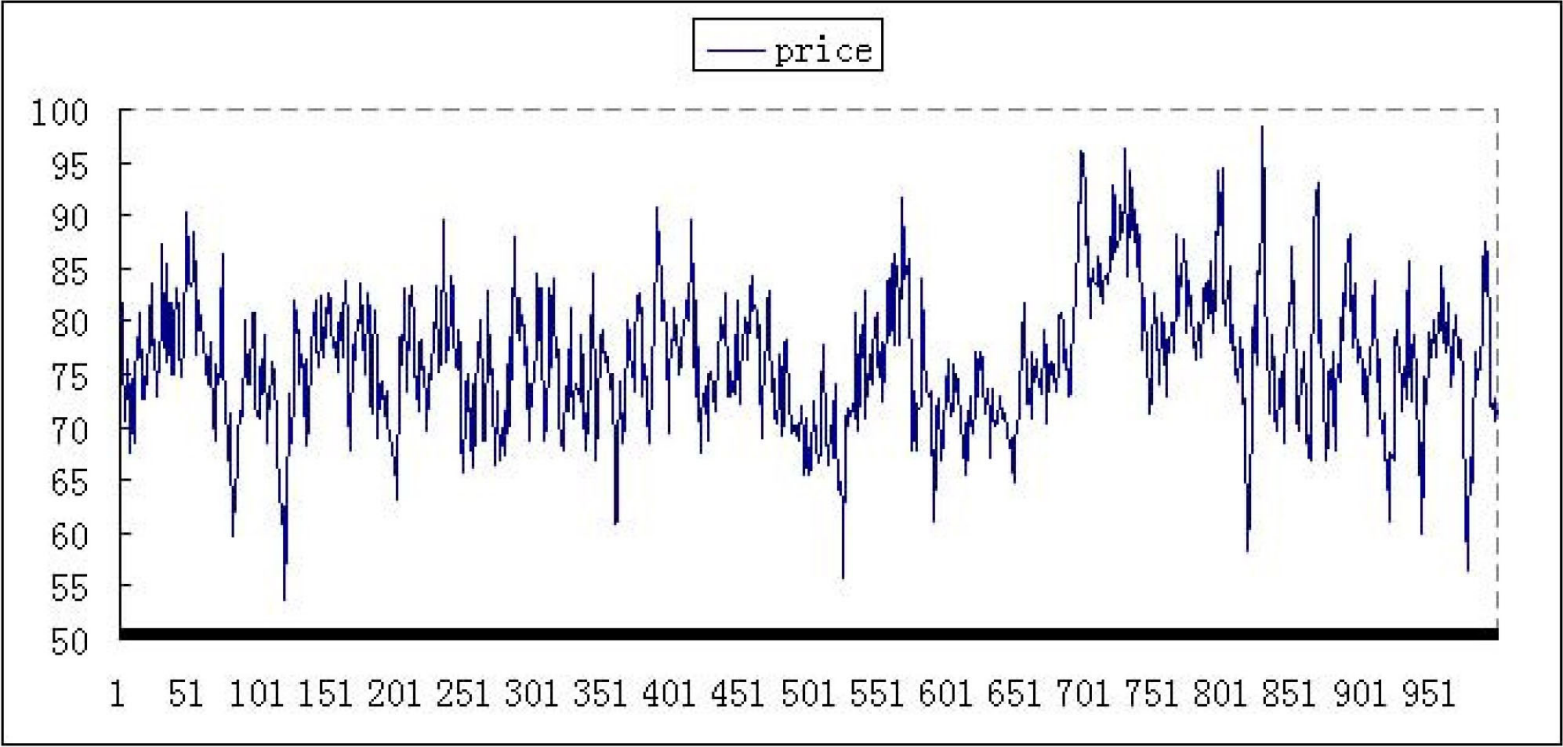
The price with 10% zero-intelligence agents at weekly frequency.

**Fig 7:**
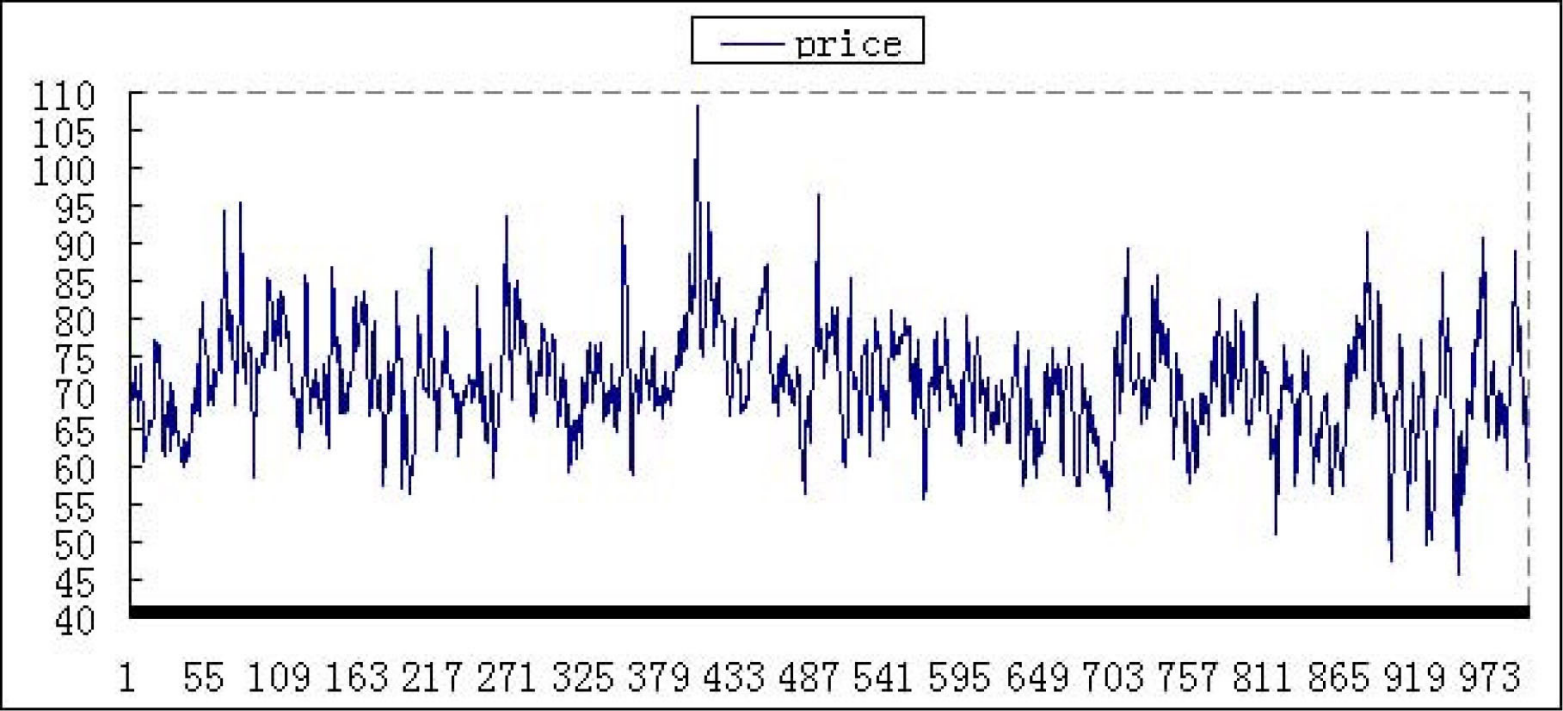
The price with 10% less-intelligence agents at weekly frequency.

In this case, the performance with zero-intelligence or less-intelligence improved significantly, and the price fluctuates at a certain range. There is no minimum price or abnormal high value in the market with zero-intelligence or less-intelligence. For example, price performances are shown in Figs 6 and 7. The ordinate is stock price, and the abscissa is time after 250000 periods.

**Fig 8:**
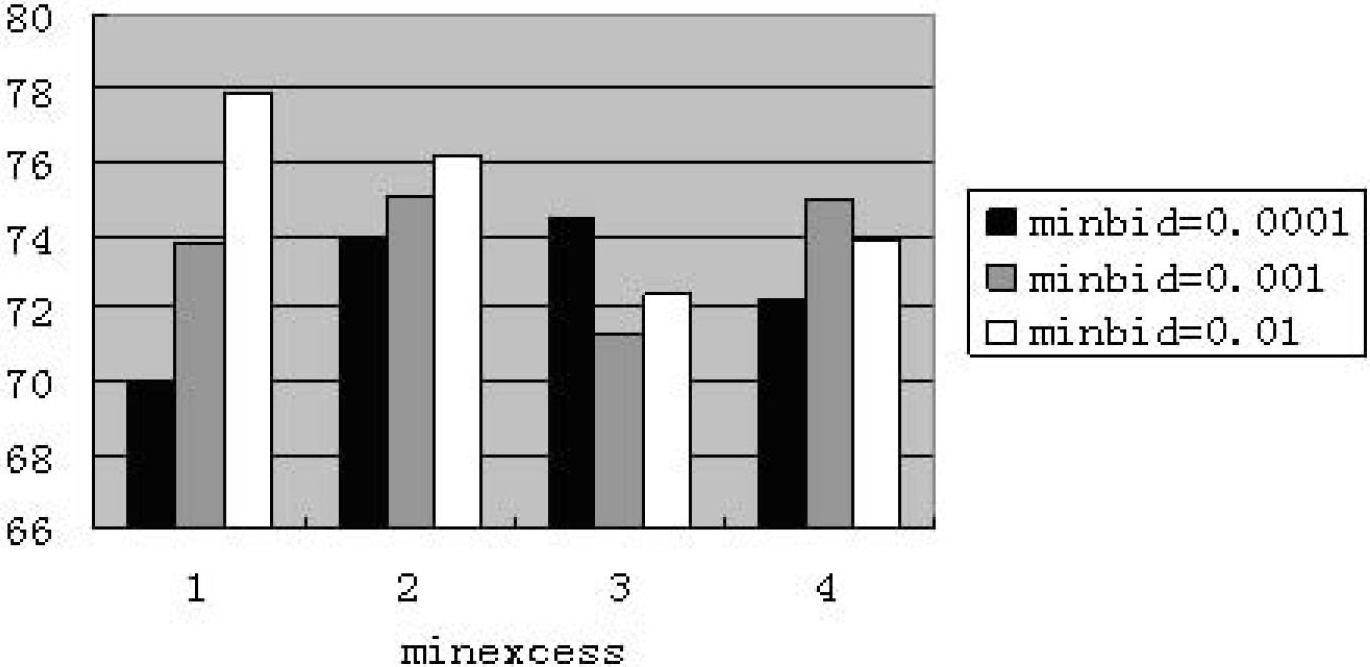
Average price for zero-intelligence at weekly frequency (The abscissa denotes four cases of “minexcess”; the ordinate denotes the percentage of price reaching the value of “minexcess”.).

**Fig 9:**
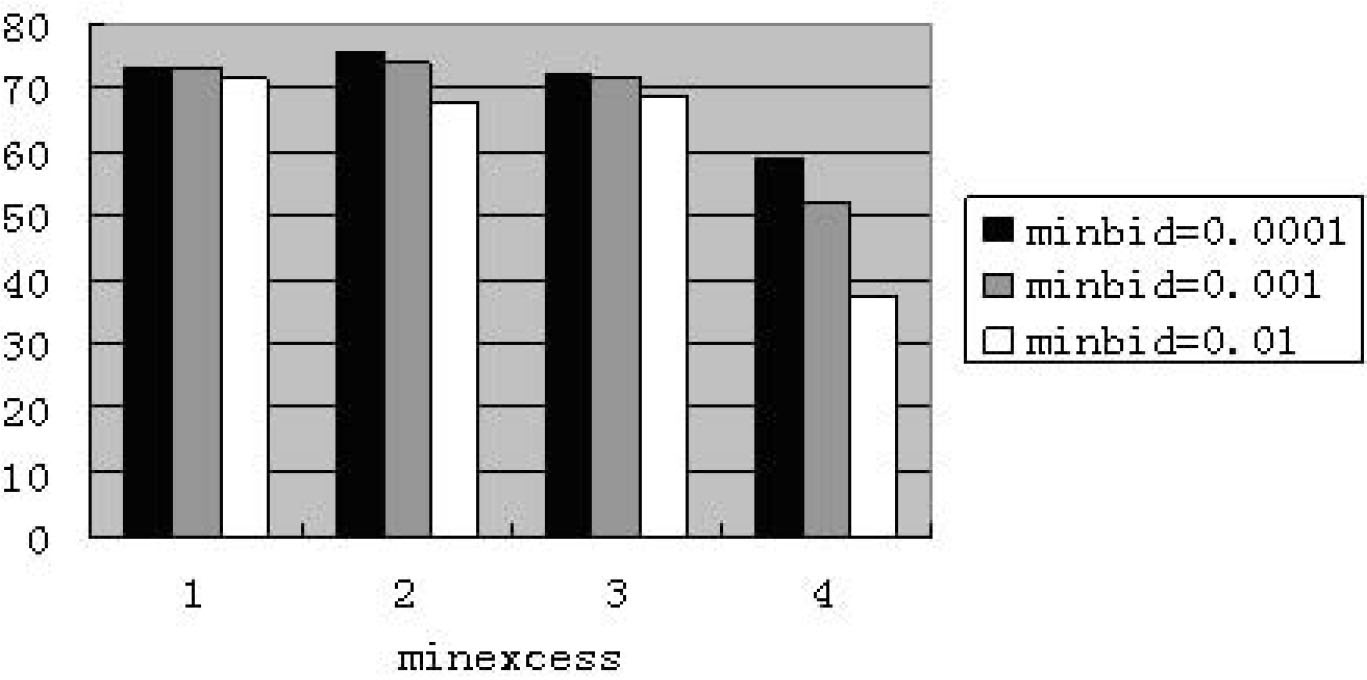
Average price for less-intelligence at weekly frequency (The abscissa denotes four cases of “minexcess”; the ordinate denotes the percentage of price reaching the value of “minexcess”.).

Figs 8 and 9 respectively illustrate the average price of market with zero-intelligence and less-intelligence.

The average price is lower than others when “minexcess” equals 1 in the market with less-intelligence. It is because a small range of minimum price appears as “minexcess” being 1 and “minbid” set as 0.01, 0.001. These minimum values pull down the average price and make the simulation invalid.

The performance of data at the daily frequency is similar. Market including 10% zero-intelligence or 10% less-intelligence (except for small part of experiments) separately performs well under the condition “maxbid” being 1 at both frequencies. In order to verify the further performance of the model with these combined parameters, we extend the experiment to 10 times and lengthen the running periods to 100000.

Now we add another five experiments for each parameter combination. S1 and S2 Tables show average standard deviation of the price at the weekly frequency in the market with zero-intelligence or less-intelligence. The results of former experiments are recorded, and the ones of following experiments are below, i.e., 7.05 is the standard deviation of five former experiments, and 9.2 is the standard deviation of following five former experiments. The results implicate that the volatility of price has very strong relation with the parameters (“minexcess” and “minbid”). The average of Std.Dev volatiles is between 6 and 8. For most parameter combination, experiments run steadily and show normal price volatility. Among these, there are three results exceeding value 9. They are produced by (minbid, minexcess) set as (0.0001, 0.1), (0.0001, 0.001) and (0.001, 0.0001). In these cases, small-range of minimum value emerges in only one experiment, which magnifies the volatility.

For market including less-intelligence, the average volatility scale of Std.Dev is similar than the market with zero-intelligence. Experiments with most of parameter combination also run steadily and show normal price volatility. Small-range of minimum value emerges in experiments with (minbid, minexcess) set as (0.001, 0.1) and (0.001, 0.1), which enlarges the volatility. Then, we lengthen the running periods of experiments with certain parameter combinations to verify the stability of the system. After first 100000 periods, the next 1000 sample is taken down and the averages of their standard deviation and price are calculated. At the same time, we also monitor the price movement roughly during the running time to ensure it is normal. Moreover, we find that the scale of standard deviation of volatility and average price are similar than those of produced by samples after 250000 periods in most parameter combinations as “maxbid” being 1. The results of experiments are really steadily which are presented in S3 Table.

In summary, the value of “maxbid” has a significant influence on the experimental results. According to above results, “maxbid” is setting as 1. “minbid” and “minexcess” have some kind of impact on simulation under this condition, but the pattern is not clear. Without more abnormal high price appears, we choose the weakest restriction, 0.0001, for “minbid”. As for “minexcess”, we use the value of the original model, 0.01. Under this parameter combination (maxbid = 1, minbid = 0.0001, minexcess = 0.01), we gradually perform further experiments.

### Critical parameter to keep the model steady

Under the parameter combination above mentioned, we add more zero-intelligence or less-intelligence in the model to search for the critical percentages which ensure the model to run steadily. The value in parentheses is the times of experiments for validating this parameter (S4–S7 Tables). To ensure the model stability, the maximum percentage of the zero-intelligent agent which could be added to the market is 33.3%, and the data for less-intelligence is 16.7%. Some level of abnormal high values emerges in the market that contains less-intelligent ones.

Completely random acts of bidding and offering of zero-intelligence offset each other at certain extent; it mitigates the overall impact on the market. As for less-intelligence, their forecasting rules tend to be similar to the evolution based on fitness in general. They choose one from the set to guide their trades, the more likely similar act magnify the abnormal price movement in total. That may be the reason for more zero-intelligent agents could be added to the model.

### Agents with different level of intelligence

We test model including agents with different level of intelligence under the parameter combination given in subsection 3.3.2 and the maximum percentage of adding restriction given in subsection 3.3.3 to produce results more reasonable comparing with real data.

The parameter of the experiment is the percentage of agents with different intelligent level. For example, (20%, 1, 2) denotes 20% of non-intelligent agents and the proportion of zero-intelligence to less-intelligence is 1:2. The experiment runs 10 times for each kind parameter. If the data rises with a wide range of minimum values or abnormal high one, we do not list the statistical features of data produced by this related parameter in S8 Table.

S9 Table shows that when the ratio of zero-intelligence agents to less-intelligence ones is set as 2:1, and the total of them taking 20% for all agents, the results have an improvement. Yield series and the square of yield series show no first-order auto-correlation, just like statistical features of data produced by real data, and the value of kurtosis is still small.

The performance of the model is improved under the parameter of (10%, 2, 1) and (20%, 5, 1). Yield series presents no first-order auto-correlation and square of yield series shows auto-correlation significantly. Moreover, Std.Dev is still well fit with real data as the original model, and the value of kurtosis is also small. The experiments show that the more less-intelligent agents are appended in the model, the more possible to bring the model damaging influence. It is consistent with the conclusion in subsection 3.3.3.

### Testing model before and after financial crisis

In order to calibrate our heterogeneous agent model, we choose some real financial market data as the benchmark. All of the chosen data are main stock indexes of Chinese stock market and other main stock markets around the world. Specifically, since stock markets sustained great macroeconomic turbulence due to US subprime crisis which later led to a global financial crisis, the stock markets are divided into two parts: before the financial crisis and after it. So, a stock market index is divided into before financial crisis and after it. US subprime crisis began to show up since the Feb. 2007, while its impact did not reach Chinese stock markets until Oct. 2007. So, we choose the data from 2006/09/01 to 2007/09/30 to represents the period before finance crisis and the data from 2008/09/01 to 2009/09/30 to represents the period after it.

Chinese stock market indexes are Shanghai Composite Index (code: 000001), Shenzhen Component Index (code: 399001), Shenzhen A Index (code: 399107) and Shanghai and Shenzhen 300 Index (code: 399300). Our experiments intercept the data from 2006/09/01 to 2007/09/30 and abandon the rest of date. Other major stock market indexes are all over the world: Dow Jones Industrial Average Index (code: DJI), NASDAQ Composite Index (code: NASDAQ), Standard and Poor’s Composite Index (code: S&P500), Hang Seng Index (code: HSI001), Kingdom FTSE 100 (GBP) (code: GBPFTSE100) and Nikkei 225 Index (code: Nikkei 225). The experiments intercept the data from 2008/09/01 to 2009/09/30 and abandon the rest of date. The frequency of the data is set as day and week, and statistical features about the first-order autocorrelation, kurtosis, standard deviation of yield series and the first-order autocorrelation of yield square (cluster volatility) are calculated and recorded by S10–S12 Tables.

Then an optimal modified model before the financial crisis is proposed and the statistical results are obtained for comparison. As for daily frequency, the optimal proportion for evolving speeds is 1: 2: 3, the optimal proportion for strategy size is 1: 1: 3 and the optimal proportion for utility function is 1: 1. As for weekly frequency, the optimal proportion for evolving speeds is 1: 3: 2, the optimal proportion for strategy size is 2: 2: 1 and the optimal proportion for utility function is 1: 2. For each experiment, the market is run for 5000 time periods in advance to allow sufficient learning of agents. The time series of next 300 samples are recorded. The statistical results are summarized in Table 14.

**Table 14:** Statistical results of the optimal performed model before financial crisis-daily frequency.

I = 1	Evolving Speeds	Strategy Size	Utility Function
Parameter	1: 2: 3:	1: 1: 3	1: 1
Auto-correlation	0.027		
Kurtosis	2.968		
Std.Dev	0.0250		
Square–auto	-0.005		

Now we compare the results of the optimal modified model before the financial crisis and the statistical results of the real stock market after the financial crisis. As for yield series, the first-order auto-correlation in Chinese four indexes is between 0.018 and 0.057. There is no first-order auto-correlation in Shanghai and Shenzhen index at any frequency at 95% confidence level. While we look into foreign countries stock market indexes, the situation changed a little. At daily frequency, the first-order auto-correlation of six indexes is between -0.142 and 0.078. NASDAQ and Nikkei 225 Indexes show auto-correlation in some extent. The first-order auto-correlation about yield series is 0.027 in the optimal modified model before the financial crisis, which smaller than most of the real index except Shanghai and Shenzhen 300 Index (code: 399300).

As for the value of kurtosis, all the Chinese stock market real data’s kurtoses are below 4.4, and kurtoses of other markets fluctuate between 3.1 and 8.2. However, the optimal modified model before financial crisis only produces kurtosis of 2.97, which is much smaller than the results of real markets.

As for standard deviation, the optimal modified model before financial crisis performs well. The square of yield series produced by the modified model is smaller than the entire real stock index.

At weekly frequency, Table 15 shows that the first-order auto-correlation about yield series in Chinese stock market fluctuates between -0.018 and -0.038, the market presents no auto-correlation. While the optimal modified model after financial crisis produces negative first-order auto-correlation about yield series. As to the value of kurtosis, the results are all bigger than 2.9 in Chinese stock market, the foreign stock markets have an average value of kurtosis about 5.49, the modified model, which has the kurtosis of 2.65, smaller than the real market index. The optimal modified model before the financial crisis failed to replicate the statistical results of the real stock market due to the financial crisis. In next stage, the experiments try to better simulate the real market data after financial crisis by adding heterogeneous agents from different aspects using the modified structure of ASM.

**Table 15:** Statistical results of the optimal performed model before financial crisis -weekly frequency.

I = 5	Evolving Speeds	Strategy Size	Utility Function
Parameter	1: 3: 3	2: 2: 1	1: 2
Auto-correlation	-0.254		
Kurtosis	2.648		
Std.Dev	0.0470		
Square–auto	0.043		

### The optimal modified model before financial crisis

The Shanghai and Shenzhen 300 Index (code: 399300) is chosen to represent the real market data. The representative real market data, the data produced by original model and the optimal modified models before the financial crisis are summarized in Tables 16 and 17.

**Table 16:** Comparison of daily frequency.

I = 1	399300	Original model	Original model	Evolving Speeds	Strategy Size	Utility Function
Parameter	None	K = 1000	K = 250	1: 2: 3	1: 1: 3	1: 1
Auto-correlation	-0.034	0.299	-0.044	-0.071	-0.052	-0.086
Kurtosis	5.801	11.752	6.055	3.430	3.055	3.406
Std.Dev.	0.0213	0.0235	0.0277	0.0254	0.0251	0.0233
Square -auto	0.136	0.413	0.185	-0.094	0.104	-0.082

**Table 17:** Comparison of weekly frequency.

I = 5	399300	Original model	Original model	Evolving Speeds	Strategy Size	Utility Function
Parameter	None	K = 1000	K = 250	1: 3: 3	2: 2: 1	1: 2
Auto-correlation	-0.105	-0.167	-0.052	-0.087	-0.074	-0.057
Kurtosis	4.174	10.140	4.463	2.515	4.782	3.252
Std.Dev.	0.0394	0.0589	0.0628	0.0574	0.1111	0.0510
Square -auto	-0.159	0.309	0.121	-0.061	0.275	0.089

For daily frequency, the optimal of all the three experimental data of modified model well fit the representative real data in auto-correlation about yield series and square of yield series. Especially, compared to the original model, the value of kurtosis is the most improved norm in modified model. In the original model, the value of kurtosis can reach as high as 11.75 at the daily frequency and 10.14 at the weekly frequency, which seriously deviates from the average level of the value of kurtosis in real stock market. For weekly frequency, the most significant improvement is in the auto-correlation about square of yield series. The original model gets the result of 0.309 when *K* = 1000, 0.121 when *K* = 250, both are too high compared to the real market. In the modified model, the result is between -0.061 and 0.275, closer to the real stock market.

### The optimal modified model after financial crisis

The Shanghai and Shenzhen 300 Index (code: 399300) and Dow Jones Industrial Average Index (code: DJI) are chosen to represent the real market data. The representative real market data and the optimal performed of modified models after the financial crisis are summarized as below:

At daily frequency, the optimal of all the three experimental data of modified model well fit the representative real data in auto-correlation about yield series and square of yield series. Especially, compared to the original model, the value of kurtosis is the most improved norm in modified model. In the original model, the value of kurtosis can reach as high as 11.75 at the daily frequency and 10.14 at the weekly frequency, which seriously deviated from the average level of the value of kurtoses in real stock markets.

At weekly frequency, the most significant improvement is in the auto-correlation about the square of yield series. The original model gets the result of 0.309 when *K* = 1000, 0.121 when *K* = 250, both are too high compared to the real market. In the modified model, the result is between -0.011 and 0.131, closer to the real stock market.

Now, the optimal performed modified model will be tested. We choose the stock market index from 2009/10/01 to 2010/01/29, it is the period just following 2009/09/30. As above mentioned, we use the not only stock market index of China, but also US, England, Japan and Hong Kong, they are all financial center in the world. The statistical results of these stock markets are summarized as S13–S15 Tables.

Then we run the optimal performed of modified model and get the statistical data for comparison. As for daily frequency, Table 18 shows that the optimal proportion for evolving speeds is 1: 3: 1, the optimal proportion for strategy size is 1: 1: 3, the optimal proportion for utility function is 1: 2. As for weekly frequency, the optimal proportion for evolving speeds is 1: 2: 2, the optimal proportion for strategy size is 1: 1: 3, the optimal proportion for utility function is 1: 2. For every experiment, the market is run for 5000 time periods in advance to allow sufficient learning and early transients to die out. The time series of next 100 samples are recorded. The statistical features of data are summarized in Tables 18, 19 and 20.

**Table 18:** Statistical results of the optimal performed model after financial crisis -daily frequency.

I = 1	Evolving Speeds	Strategy Size	Utility Function
Parameter	1: 3: 1	1: 1: 3	1: 2
Auto-correlation	-0.012		
Kurtosis	4.000		
Std.Dev	0.0173		
Square–auto	-0.021		

**Table 19:** Comparison of the optimal modified model and real market -daily frequency.

I = 1	Optimal modified model	Real market
Auto-correlation	-0.012	All bigger than -0.012 except Nikkei 225
Kurtosis	4.00	2.9–3.7, a little smaller than 4.00
Std.Dev	0.017	0.016–0.018, well fit
Square–auto	-0.021	Chinese market close to -0.021, foreign market bigger than -0.021

**Table 20:** Statistical results of the optimal model after financial crisis -weekly frequency.

I = 5	Evolving Speeds	Strategy Size	Utility Function
Parameter	1: 2: 2	1: 1: 3	1: 2
Auto-correlation	-0.178		
Kurtosis	3.025		
Std.Dev	0.0485		
Square–auto	0.085		

At daily frequency, the optimal modified model produces first-order auto-correlation about yield series smaller than most of the real markets data except Nikkei 225, this shows it is still a little too ideal compared with the real stock market. The value of kurtosis is 4, while all the real data has a value of kurtosis between 2.9 and 3.7. The optimal performed of modified model exaggerates the value of kurtosis. The standard deviation value is 0.017, as the value of kurtosis in Chinese stock market fluctuates between 0.016 and 0.018, we can say the kurtosis fits well with the Chinese real market. The auto-correlation about the square of yield series also performs well comparing to the real data.

At weekly frequency, Table 21 shows that the first-order auto-correlation about yield series in Chinese stock market fluctuates between -0.271 and -0.511, the market presents negative auto-correlation, while there is no such feature in foreign stock markets. The first-order auto-correlation about yield series in our modified model is -0.178, also shows negative auto-correlation to some extended. As to the value of kurtosis, the results are all small than 2 in Chinese stock market, the foreign stock markets have an average value of kurtosis about 3, the modified model, which has the kurtosis of 3.02, fits foreign stock market better than Chinese stock market this time. As to standard deviation, the modified model performs perfectly compare with Chinese stock market. Both of them have the value of standard deviation around 0.04, a little bigger than the foreign stock market.

**Table 21:** Comparison of the optimal modified model and real market -weekly frequency.

I = 5	Optimal modified model	Real market
Auto-correlation	-0.178	Chinese market: -0.271 to -0.511, stronger negative auto-correlation than modified model
Kurtosis	3.025	Chinese market: 2, foreign market: 3
Std.Dev	0.0485	Chinese market: 0.04. foreign market: smaller than 0.04
Square–auto	0.085	No regular pattern

## Conclusion

In this paper, a heterogeneous model is proposed by changing the matching and choosing the policy of forecasting. On the view of agents about financial markets, heterogeneity is appending to agents by four different modes: evolving speed, size of strategy pool, aversion level of risk and intelligent level. The experimental results are compared with the data produced by real financial markets.

In the artificial market of intelligent agents, all three experiments, with different evolving speed, size of strategy pool and risk aversion, results fit the representative real data in auto-correlation about yield series and square of yield series at the daily frequency. About weekly frequency, the optimal of all the three experimental data possess a lower level of first-order auto-correlation about yield series than the original model. And the data of experiments of different size of strategy pool and risk aversion well fit the real data in the square of yield series; meanwhile, data of the first experiment presents little of that. In that scenario, the modified model does not solve the problem that the experimental data still possess first-order auto-correlation about yield series at the weekly frequency, and the value of kurtosis is small than the real data.

Two new kinds of agents are employed, the zero-intelligent and the less-intelligent agent. Agents with different intelligence, the original model fails to simulate the normal price. To solve this problem, we append and modify some parameters, “minbid” and “maxbid” for agents and “minexcess” for specialists adjusting the price. Experiments show that “maxbid” has a significant influence on the results and set it to be 1. The “minbid” is chosen the level of weakest restriction and the “minexcess” is reserved original value. Under this parameter combination, the maximum percentage of zero-intelligence and less-intelligence can be appended to model is 33.3% and 16.7% respectively to maintain the stability of the model. And then, the percentages of these three agents are found to produce results fit the real data better than the original model. The setting of heterogeneity in intelligence level is closer to real situations.

In order to validate the novel model can find the structure change of stock market by the financial crisis; the real stock markets are divided into two parts, one before the financial crisis and the other after it. Firstly, the data are sampled before the financial crisis. Experiments results fit the representative real data well, with different evolving speed, size of strategy pool and risk aversion. Consider every experiment’s optimal parameters, an optimal performed modified model before the financial crisis is presented. For daily frequency, the optimal parameters are Evolving Speeds of 1:2:3, Strategy Size of 1:1:3, Utility of 1:1. For weekly frequency, the optimal parameters are Evolving Speeds of 1:3:2, Strategy Size of 2:2:1, Utility of 1:2. Then the optimal modified model is used before the financial crisis to simulate the real stock markets after the financial crisis, the results are disappointing. The optimal model before financial crisis produces auto-correlation of yield series smaller than most of the real stock markets, it also produces a smaller value of kurtosis than all of the real markets. Then, further modification of the model benefits to find the optimal modified model after the financial crisis. Experiments show that when using the Evolving Speeds of 1:3:1, Strategy Size of 1:1:3, Utility of 1:2 at daily frequency and Evolving Speeds of 1:2:2, Strategy Size of 1:1:3, Utility of 1:2 at weekly frequency, the model produces auto-correlation of yield series neither too big or too small, the value of kurtosis similar than those of real markets, and also reasonable standard deviation and auto-correlation of the square of yield series, close to the real market after financial crisis. Finally, if the optimal model can produce statistical features of data more closely to those of the real stock market after the financial crisis, a comparison is made between the simulated model and the real stock market between the period of 2009/10/31 and 2010/01/29. The modified model after financial crisis produces standard deviation and auto-correlation of the square of yield series perfectly match with those of real stock markets. Although the first-order auto-correlation of yield series is smaller than those of real markets data at daily frequency and that the value of kurtosis is bigger than those of real markets data at daily frequency, which means the modified model is still a little too ideal compared with the real stock market, and the optimal modified models already make a good replication of the real stock markets. Comparing the models between before financial crisis and after it, the structure change is obvious, which validates intuitional investigations.

## Supporting information

S1 Software ASMforPLos. (ZIP)

S1 Dataset DATASET. (ZIP)

S1 Table Average Std.Dev of price for zero-intelligence at weekly frequency. (DOCX)

S2 Table Average Std.Dev of price for zero-intelligence at daily frequency. (DOCX)

S3 Table Std.Dev and price for long-period at weekly frequency. (DOCX)

S4 Table Zero-intelligence agents at weekly frequency. (DOCX)

S5 Table Zero-intelligence agents at daily frequency. (DOCX)

S6 Table Less-intelligence agents at weekly frequency. (DOCX)

S7 Table Less-intelligence agents at daily frequency. (DOCX)

S8 Table Zero-intelligence and less-intelligence agents at weekly frequency. (DOCX)

S9 Table Zero-intelligence and less-intelligence agents at daily frequency. (DOCX)

S10 Table Statistical results of Chinese real stock index-after financial crisis. (DOCX)

S11 Table Statistical results of American real stock index -after financial crisis. (DOCX)

S12 Table Statistical results of Hong Kong, Great British and Japanese real stock market index. (DOCX)

S13 Table Statistical results of Chinese real stock index. (DOCX)

S14 Table Statistical results of American real stock index. (DOCX)

S15 Table Statistical results of Hong Kong, Great British and Japanese real stock market index. (DOCX)
