# Supplementary material for: A heterogeneous artificial stock market model can benefit people against another financial crisis
Source: PLoS One. 2018 Jun 18;13(6):e0197935. doi: 10.1371/journal.pone.0197935 (PMC6005484; doi:10.1371/journal.pone.0197935)
Supplement: S1 Table — (DOCX) [file pone.0197935.s003.docx]

**S1 Table Average Std.Dev of price for zero-intelligence at daily frequency**

|  | minexcess  0.1 | minexcess  0.01 | minexcess  0.001 | minexcess 0.0001 |
| --- | --- | --- | --- | --- |
| Minbid  0.0001 | 7.05 | 6.51 | 7.34 | 7.03 |
|  | 9.2 | 7.04 | 10.41 | 6.88 |
| Minbid  0.001 | 6.44 | 6.84 | 7.13 | 7.36 |
|  | 7.27 | 7.24 | 7.18 | 9.78 |
| Minbid  0.01 | 6.28 | 6.93 | 7.21 | 7.04 |
|  | 6.25 | 6.73 | 6.45 | 6.59 |
