# Supplementary material for: A heterogeneous artificial stock market model can benefit people against another financial crisis
Source: PLoS One. 2018 Jun 18;13(6):e0197935. doi: 10.1371/journal.pone.0197935 (PMC6005484; doi:10.1371/journal.pone.0197935)
Supplement: S2 Table — (DOCX) [file pone.0197935.s004.docx]

**S2 Table Average Std.Dev of price for zero-intelligence at weekly frequency**

|  | minexcess  0.1 | minexcess  0.01 | minexcess  0.001 | minexcess  0.0001 |
| --- | --- | --- | --- | --- |
| Minbid  0.0001 | 7.01 | 6.76 | 7.44 | 6.57 |
|  | 6.86 | 7.10 | 6.15 | 6.49 |
| Minbid  0.001 | 10.47 | 7.06 | 6.75 | 8.46 |
|  | 7.36 | 7.09 | 8.94 | 6.89 |
| Minbid  0.01 | 10.34 | 6.37 | 6.98 | 7.29 |
|  | 7.23 | 7.94 | 7.57 | 7.37 |
