# Supplementary material for: A heterogeneous artificial stock market model can benefit people against another financial crisis
Source: PLoS One. 2018 Jun 18;13(6):e0197935. doi: 10.1371/journal.pone.0197935 (PMC6005484; doi:10.1371/journal.pone.0197935)
Supplement: S3 Table — (DOCX) [file pone.0197935.s005.docx]

**S3 Table Std.Dev and price for long-period at weekly frequency**

| Minbid  minexcess | 0.0001  0.0001 | 0.0001  0.01 | 0.001  0.01 | 0.01  0.0001 | 0.01  0.001 | 0.01  0.01 |
| --- | --- | --- | --- | --- | --- | --- |
| Zero-intelligence Std.Dev | 7.21 | 7.14 | 6.86 | 6.38 | 6. 53 | 6.78 |
| Zero-intelligence Price | 72.74 | 74.18 | 73.87 | 76.43 | 74.86 | 75.60 |
| Less-intelligence Std.Dev | 6.04 | 6.69 | 6.39 | 6.66 | 7.11 | 7.05 |
| Less-intelligence price | 77.10 | 77.03 | 72.91 | 78.15 | 73.13 | 72.88 |
