# Supplementary material for: A heterogeneous artificial stock market model can benefit people against another financial crisis
Source: PLoS One. 2018 Jun 18;13(6):e0197935. doi: 10.1371/journal.pone.0197935 (PMC6005484; doi:10.1371/journal.pone.0197935)
Supplement: S4 Table — (DOCX) [file pone.0197935.s006.docx]

**S4 Table Zero-intelligence agents at weekly frequency**

| percentage | 50%（5） | 40%（5） | 33.3%（10） | 30%（10） |
| --- | --- | --- | --- | --- |
| Price | 6.57 | 29.69 | 54.95 | 68.25 |
| Std.Dev | 3.98 | 7.6 | 7.69 | 6.93 |
| Reaching minimum value | 15.4% | 8.8% | 0% | 0% |
