# Supplementary material for: A heterogeneous artificial stock market model can benefit people against another financial crisis
Source: PLoS One. 2018 Jun 18;13(6):e0197935. doi: 10.1371/journal.pone.0197935 (PMC6005484; doi:10.1371/journal.pone.0197935)
Supplement: S5 Table — (DOCX) [file pone.0197935.s007.docx]

**S5 Table Zero-intelligence agents at daily frequency**

| Percentage | 50%（5） | 40%（5） | 33.3%（10） | 30%（10） |
| --- | --- | --- | --- | --- |
| Price | 13.34 | 30.29 | 53.78 | 55.89 |
| Std.Dev | 3.78 | 5.13 | 5.73 | 6.71 |
| Reaching minimum value | 4.8% | 4.6% | 0% | 0% |
