# Supplementary material for: A heterogeneous artificial stock market model can benefit people against another financial crisis
Source: PLoS One. 2018 Jun 18;13(6):e0197935. doi: 10.1371/journal.pone.0197935 (PMC6005484; doi:10.1371/journal.pone.0197935)
Supplement: S6 Table — (DOCX) [file pone.0197935.s008.docx]

**S6 Table Less-intelligence agents at weekly frequency**

| Percentage | 30%（5） | 20%（5） | 16.7%（10） |
| --- | --- | --- | --- |
| Price | 114.2 | 92.43 | 73.51 |
| Std.Dev | 621.45 | 104.95 | 7.01 |
| Reaching minimum value | 9.7 | 6.9% | 0% |
| Abnormal high value | 10.4% | 1.1% | 0% |
