# Supplementary material for: A heterogeneous artificial stock market model can benefit people against another financial crisis
Source: PLoS One. 2018 Jun 18;13(6):e0197935. doi: 10.1371/journal.pone.0197935 (PMC6005484; doi:10.1371/journal.pone.0197935)
Supplement: S7 Table — (DOCX) [file pone.0197935.s009.docx]

**S7 Table Less-intelligence agents at daily frequency**

| Percentage | 30%（5） | 20%（10） | 16.7%（10） |
| --- | --- | --- | --- |
| Price | 103.23 | 64.53 | 75.62 |
| Std.Dev | 435.87 | 6.18 | 6.94 |
| Reaching minimum value | 12.1% | 0.7% | 0% |
| Abnormal high value | 4.7% | 0% | 0% |
