# Supplementary material for: A heterogeneous artificial stock market model can benefit people against another financial crisis
Source: PLoS One. 2018 Jun 18;13(6):e0197935. doi: 10.1371/journal.pone.0197935 (PMC6005484; doi:10.1371/journal.pone.0197935)
Supplement: S8 Table — (DOCX) [file pone.0197935.s010.docx]

**S8 Table Zero-intelligence and less-intelligence agents at weekly frequency**

|  | 10% 1 2 | 10% 2 1 | 20% 1 1 | 20% 2 1 | 20% 5 1 |
| --- | --- | --- | --- | --- | --- |
| Autocorrelation | -0.045 | -0.107 | -0.163 | -0.034 | -0.135 |
| Kurtosis | 4.16 | 10.78 | 3.14 | 3.59 | 3.22 |
| Std.Dev. | 0.0640 | 0.0791 | 0.0692 | 0.0707 | 0.0599 |
| Square-auto | 0.156 | 0.191 | 0.066 | 0.049 | 0.092 |
|  | 30% 7 2 | 30% 2 1 | 30% 5 4 | 40% 3 1 |  |
| Autocorrelation | -0.229 | -0.127 | -0.168 | -0.317 |  |
| Kurtosis | 3.77 | 7.27 | 3.22 | 10.90 |  |
| Std.Dev. | 0.1546 | 0.0811 | 0.0701 | 0.3487 |  |
| Square-auto | 0.176 | 0.276 | 0.124 | 0.301 |  |
