# Supplementary material for: A heterogeneous artificial stock market model can benefit people against another financial crisis
Source: PLoS One. 2018 Jun 18;13(6):e0197935. doi: 10.1371/journal.pone.0197935 (PMC6005484; doi:10.1371/journal.pone.0197935)
Supplement: S9 Table — (DOCX) [file pone.0197935.s011.docx]

**S9 Table Zero-intelligence and less-intelligence agents at daily frequency**

|  | 10% 1 2 | | 10% 2 1 | | 20% 1 1 | | 20% 2 1 | 20% 1 2 |
| --- | --- | --- | --- | --- | --- | --- | --- | --- |
| Autocorrelation | -0.041 | | -0.045 | | 0.062 | | -0.177 | -0.039 |
| Kurtosis | 3.31 | | 3.34 | | 3.11 | | 3.10 | 3.14 |
| Std.Dev. | 0.0283 | | 0.0278 | | 0.0318 | | 0.0298 | 0.0411 |
| Square-auto | 0.033 | | 0.136 | | 0.052 | | 0.105 | 0.038 |
|  | | 20% 1 5 | | 20% 5 1 | |  |  |  |
| Autocorrelation | | 0.015 | | -0.065 | |  |  |  |
| Kurtosis | | 3.17 | | 3.16 | |  |  |  |
| Std.Dev. | | 0.0486 | | 0.0274 | |  |  |  |
| Square-auto | | 0.070 | | 0.206 | |  |  |  |
