# Supplementary material for: A heterogeneous artificial stock market model can benefit people against another financial crisis
Source: PLoS One. 2018 Jun 18;13(6):e0197935. doi: 10.1371/journal.pone.0197935 (PMC6005484; doi:10.1371/journal.pone.0197935)
Supplement: S10 Table — (DOCX) [file pone.0197935.s012.docx]

**S10 Table Statistical results of Chinese real stock index-after financial crisis**

| Code | 000001(day) | 399001(day) | 399107(day) | 399300(day) |
| --- | --- | --- | --- | --- |
| autocorrelation | 0.034 | 0.050 | 0.057 | 0.018 |
| Kurtosis | 4.382 | 3.844 | 3.945 | 3.944 |
| Std.Dev | 0.0234 | 0.0251 | 0.0245 | 0.0251 |
| Square –auto | 0.177 | 0.047 | 0.071 | 0.117 |
| Code | 000001(week) | 399001(week) | 399107(week) | 399300 (week) |
| autocorrelation | 0.035 | 0.018 | 0.038 | 0.03 |
| Kurtosis | 2.977 | 3.477 | 3.562 | 3.230 |
| Std.Dev | 0.0528 | 0.0584 | 0.0573 | 0.0574 |
| Square –auto | -0.008 | -0.094 | -0.097 | -0.113 |
