# Supplementary material for: A heterogeneous artificial stock market model can benefit people against another financial crisis
Source: PLoS One. 2018 Jun 18;13(6):e0197935. doi: 10.1371/journal.pone.0197935 (PMC6005484; doi:10.1371/journal.pone.0197935)
Supplement: S11 Table — (DOCX) [file pone.0197935.s013.docx]

**S11 Table Statistical results of American real stock index -after financial crisis**

| Code | DJI (day) | NASDAQ (day) | S&P500 (day) |
| --- | --- | --- | --- |
| autocorrelation | 0.067 | -0.12 | 0.078 |
| Kurtosis | 3.562 | 5.087 | 3.849 |
| Std.Dev | 0.0081 | 0.0276 | 0.0109 |
| Square –auto | -0.136 | 0.109 | -0.201 |
| Code | DJI (week) | NASDAQ (week) | S&P500 (week) |
| autocorrelation | -0.142 | -0.071 | -0.158 |
| Kurtosis | 5.381 | 4.620 | 4.421 |
| Std.Dev | 0.0489 | 0.0523 | 0.0536 |
| Square –auto | 0.054 | 0.151 | 0.091 |
