# Supplementary material for: A heterogeneous artificial stock market model can benefit people against another financial crisis
Source: PLoS One. 2018 Jun 18;13(6):e0197935. doi: 10.1371/journal.pone.0197935 (PMC6005484; doi:10.1371/journal.pone.0197935)
Supplement: S12 Table — (DOCX) [file pone.0197935.s014.docx]

**S12 Table Statistical results of Hong Kong, Great British and Japanese real stock market index**

| Code | HSI001 (day) | GBP FTSE100 (day) | Nikkei 225 (day) |
| --- | --- | --- | --- |
| autocorrelation | -0.052 | -0.046 | -0.142 |
| Kurtosis | 6.868 | 3.133 | 3.416 |
| Std.Dev | 0.0308 | 0.0385 | 0.0193 |
| Square –auto | 0.32 | 0.009 | 0.026 |
| Code | HSI001 (week) | GBP FTSE100 (week) | Nikkei 225 (week) |
| autocorrelation | -0.057 | -0.182 | -0.09 |
| Kurtosis | 3.474 | 6.950 | 8.110 |
| Std.Dev | 0.0568 | 0.0527 | 0.0553 |
| Square –auto | 0.028 | 0.009 | 0.11 |
