# Supplementary material for: A heterogeneous artificial stock market model can benefit people against another financial crisis
Source: PLoS One. 2018 Jun 18;13(6):e0197935. doi: 10.1371/journal.pone.0197935 (PMC6005484; doi:10.1371/journal.pone.0197935)
Supplement: S13 Table — (DOCX) [file pone.0197935.s015.docx]

**S13 Table Statistical results of Chinese real stock index**

| Code | 000001(day) | 399001(day) | 399107(day) | 399300(day) |
| --- | --- | --- | --- | --- |
| Autocorrelation | -0.033 | -0.022 | -0.027 | -0.046 |
| Kurtosis | 3.349 | 3.703 | 3.661 | 3.701 |
| Std.Dev | 0.0158 | 0.0179 | 0.0180 | 0.0169 |
| Square –auto | -0.002 | 0.041 | 0.038 | 0.021 |
| Code | 000001(week) | 399001(week) | 399107(week) | 399300(week) |
| Autocorrelation | -0.511 | -0.288 | -0.271 | -0.345 |
| Kurtosis | 1.876 | 1.907 | 1.925 | 1.928 |
| Std.Dev | 0.0403 | 0.0450 | 0.0449 | 0.0430 |
| Square –auto | 0.091 | -0.227 | -0.303 | -0.028 |
