# Supplementary material for: A heterogeneous artificial stock market model can benefit people against another financial crisis
Source: PLoS One. 2018 Jun 18;13(6):e0197935. doi: 10.1371/journal.pone.0197935 (PMC6005484; doi:10.1371/journal.pone.0197935)
Supplement: S14 Table — (DOCX) [file pone.0197935.s016.docx]

**S14 Table Statistical results of American real stock index**

| Code | DJI (day) | NASDAQ (day) | S&P500 (day) |
| --- | --- | --- | --- |
| Autocorrelation | -0.09 | -0.044 | -0.099 |
| Kurtosis | 3.214 | 3.242 | 3.164 |
| Std.Dev | 0.0094 | 0.0110 | 0.0103 |
| Square –auto | 0.141 | 0.005 | 0.132 |
| Code | DJI (week) | NASDAQ (week) | S&P500 (week) |
| Autocorrelation | 0.015 | -0.027 | -0.002 |
| Kurtosis | 2.900 | 2.501 | 2.510 |
| Std.Dev | 0.0202 | 0.0256 | 0.0231 |
| Square –auto | -0.084 | -0.086 | -0.051 |
