# Supplementary material for: A heterogeneous artificial stock market model can benefit people against another financial crisis
Source: PLoS One. 2018 Jun 18;13(6):e0197935. doi: 10.1371/journal.pone.0197935 (PMC6005484; doi:10.1371/journal.pone.0197935)
Supplement: S15 Table — (DOCX) [file pone.0197935.s017.docx]

**S15 Table Statistical results of Hong Kong, Great British and Japanese real stock market index**

| Code | HSI001 (day) | GBP FTSE100 (day) | Nikkei 225 (day) |
| --- | --- | --- | --- |
| Autocorrelation | 0.062 | -0.209 | -0.002 |
| Kurtosis | 3.342 | 2.927 | 3.350 |
| Std.Dev | 0.0142 | 0.0110 | 0.0128 |
| Square –auto | 0.275 | -0.133 | 0.082 |
| Code | HSI001 (week) | GBP FTSE100 (week) | Nikkei 225 (week) |
| Autocorrelation | -0.127 | -0.009 | -0.053 |
| Kurtosis | 1.930 | 2.121 | 4.664 |
| Std.Dev | 0.0356 | 0.0219 | 0.0343 |
| Square –auto | 0.125 | -0.294 | 0.012 |
